# Evaluation of fetal congenital persistence of the left superior vena cava using fetal MR

**DOI:** 10.1186/1532-429X-17-S1-P224

**Published:** 2015-02-03

**Authors:** Ming Zhu

**Affiliations:** Radiology Dept, Shanghai Children’s Medical Center, Shanghai, China

## Background

Ultrasound is the first choice to visualize the fetal cardiac defects. MRI is unaffected by special maternal conditions such as obesity and oligohydramnios, which impair sonographic visualization. The aim of this study was to analyze if fetal cardiac MRI can give more diagnostic information when without special conditions.

## Methods

Between June 2005 and April 2014, 58 fetuses with LSVC which confirmed by postnatal imaging were evaluated using fetal echo and MRI in our hospital. MRI was performed using 1.5T unit. MRI was performed at 18 to 38 weeks' gestation (mean 26.5 weeks). Imaging sequences included a multiplanar SSFP, non-gated cine SSFP and SS-TSE sequence.

## Results

The 58 cases of fetal LSVC detected by fetal MRI represents 1.23% of all fetal MRI examinations performed in our hospital (58/4726). All the 58 cases were correctly diagnosed by MR imaging but only 40 cases (69%) were correctly diagnosed by fetal ultrasound.

Of the 18 cases which did not be correctly diagnosed by fetal ultrasound, 8 were sent to perform fetal MRI for head or chest malformation. Before fetal MRI they only did general fetal ultrasound and never did fetal echocardiography. 9 did fetal echocardiography before fetal MRI. But the doctors are from small hospital and no enough experience. Only one case which did not be correctly diagnosed was performed fetal echocardiography by experienced doctors and this case first time performed fetal echocardiography was at 34 weeks gestation. After the fetal cardiac MRI examination, all the 18 cases were performed fetal echocardiography again and all can find persistent LSVC.

In the 40 cases correctly diagnosed by fetal ultrasound, two cases LSVC were correctly diagnosed using fetal echocardiography by experienced doctors but fetal MRI found bridging vessels which did not found by echocardiography. In another two cases (not include the 58 cases) LSVC was diagnosed by fetal echocardiography but fetal cardiac MRI found it was really a retroesophageal left brachiocephalic vein.

## Conclusions

This data means that radiologist must very carefully pay attention to great vessels if the patients were not performed a good fetal echocardiography. Our data also means that though LSVC can be almost 100% correctly diagnosed using fetal echocardiography by experienced doctors, at least in some cases, fetal cardiac MRI still can provide some additional important information. The fetal MR imaging of the transverse view of aortic arch is very easy to obtain and easy to read. LSVC can easily get important clues at this view.

According our experience, if in a country such as China the fetal echocardiography is not a routine examination or the doctors who perform fetal echocardiography are not all good enough, the radiologists must pay more attention to the signs of LSVC.

